# Overview of research on *Bombyx mori* microRNA

**DOI:** 10.1093/jis/14.1.133

**Published:** 2014-10-06

**Authors:** Xin Wang, Shun-ming Tang, Xing-jia Shen

**Affiliations:** Key Laboratory of Silkworm and Mulberry Genetic Improvement, Ministry of Agriculture, Jiangsu University of Science and Technology, Zhenjiang City 212018 Jiangsu Province, People’s Republic of China

**Keywords:** expression profiling analysis, microarray technology, prediction, review, silkworm, target gene

## Abstract

MicroRNAs (miRNAs) constitute some of the most significant regulatory factors involved at the post-transcriptional level after gene expression, contributing to the modulation of a large number of physiological processes such as development, metabolism, and disease occurrence. This review comprehensively and retrospectively explores the literature investigating silkworm, *Bombyx mori* L. (Lepidoptera: Bombicidae), miRNAs published to date, including discovery, identification, expression profiling analysis, target gene prediction, and the functional analysis of both miRNAs and their targets. It may provide experimental considerations and approaches for future study of miRNAs and benefit elucidation of the mechanisms of miRNAs involved in silkworm developmental processes and intracellular activities of other unknown non-coding RNAs.

## Introduction

MicroRNAs (miRNAs) represent an abundant family of non-protein-coding small RNA (ncRNA) molecules and play crucial roles in the regulation of both plant and animal developmental processes. To date, some studies related to miRNAs of the economically important silkworm, *Bombyx mori* L. (Lepidoptera: Bombicidae), have been conducted, focusing on identification, expression analysis, and prediction of function. In June 2013, miRBase 20, consisting of 24,521 entries from 206 different species, was released, and of these, 567 entries represented mature miRNAs from *B. mori.* This article summarizes current research approaches employed for the identification and prediction of functions of *B. mori* miRNAs. Data related to *B. mori* miRNAs were obtained from PubMed and the China National Knowledge Infrastructure (CNKI).

### Computational prediction of *B. mori* miRNAs

In general, miRNAs show high sequence conservation across species and are produced from a primary stem-loop structure in the nucleus, which are particularly important features of miRNAs. Accordingly, the first step of using bioinformatics software to predict new miRNAs is to conduct a homology search of the whole *B. mori* genome sequence in light of foregone pri-miRNA This generally results in a large number of sequences, which are then further screened through RNA secondary structure analysis, prediction software analysis, and dynamics analysis. Tong et al. (2006) conducted a homology search to identify homologs of previously validated miRNAs from the *B. mori* genome and identified 24 potential miRNA genes. Yu et al. (2008a) identified 114 non-redundant conserved miRNAs and 148 novel putative miRNAs from the *B. mori* genome with an elaborate forecasting system on the basis of the sRNA loop and known structural characteristics of animal pri-miRNAs. Yao (2008) employed computational and experimental RNomics to predict and validate *B. mori* miRNAs based on the sequence conservation of mature miRNAs and their precursors displaying hairpin structures, which resulted in the identification of 62 potentially conserved miRNAs. Forty-one conserved miRNAs were identified by adopting a computational homology search approach, some of which were further selected, and their identities were confirmed experimentally ( Cao et al. 2008 ). Based on the conservation of miRNA sequences, using a computational homology search based on a genomic survey sequence analysis, Huang et al. (2010) identified and described 16 novel miRNAs. Furthermore, He et al. (2008) used a combination of a computational method based on sequence homology searches and experimental identification based on microarray assays and northern blotting. Through this combined approach, 46 miRNAs, 21 plausible miRNAs, and a novel small RNA were identified in *B. mori.* Among those identified, there were 12 pairs of miRNAs and miRNA*s ( He et al. 2008 ). In other applications of miRNA predictive bioinformatics, Liu et al. (2008) used miRscan and PatScan algorithms and predicted 120 iRNA genes on the 6x and 9x genome assembly of *B. mori* based on sequence conservation and structural similarity to known miRNAs.

### Identification and expression of *B. mori* miRNAs

Microarray technology is a systematic and comprehensive technique for functional genomic investigations. Based on the principle of molecular hybridization, microarray techniques can be used to measure changes in gene expression levels among different samples simultaneously. Microarrays also provide information about the expression pattern of one gene, or a group of genes, and its relationship to other gene expression patterns. The endogenous expression of miRNAs in BmN cells was identified by microarray analysis, and 73 miRNAs were validated ( Yang 2012 ). Illumina/Solexa sequencing is a second-generation sequencing technology, which is primarily based on synthesis sequencing. Zhang et al. (2009) conducted a large-scale screening for miRNA genes in *B. mori* using synthesis deep sequencing and confirmed the presence of 354 miRNA genes using miRNA microarrays for all developmental stages, including egg, larval, pupal, and adult stages. Similarly, from 2,227,930 synthesis sequence tags, 3750 miRNA candidate genes were identified through a computational pipeline combining RNAfold and triplet SVM algorithms ( Zhou 2008 ). Using a large-scale Solexa sequencing technology, Liu et al. (2010b) validated 257 unique miRNA genes, including 202 novel and 55 previously reported genes, corresponding to 324 loci in the *B. mori* genome. Furthermore, in a study conducted at the Centre of Excellence for Genetics and Genomics of Silkmoths, Singh et al. (2010) identified four *B. mori* nucleopoly-hedrosis virus (BmNPV)-encoded miRNAs by using a combination of *in silico* and experimental methods. Another experimental method commonly used for recognizing new miRNAs is sequencing and construction of cDNA libraries enriched in miRNAs. Cai et al. (2010) obtained more than four million useful sequences from a library, which was constructed from a mixture of 14 RNA samples from different developmental stages of *B. mori,* by using a high-throughput sequencing method for miRNA identification. Using an elaborate screening protocol, they identified 287 novel candidate miRNAs, of which 59 miRNA and miRNA-stars (miRNA*) sequences were predicted. Researchers from Oklahoma State University generated small RNA libraries from feeding larvae, spinning larvae, pupae, and adults of *B. mori,* and obtained ∽2.5 million reads of 18-30 nucleotides. Subsequent sequence analysis identified 101 homologs of conserved miRNAs, 14 species-specific miRNAs, and two anti sense miRNAs in the *B. mori* genome ( Jagadeeswaran 2010 ).

Microarray is an effective technique for high-throughput genetic screening, which can authenticate all expression levels of known miRNAs in a short period and even detect expression levels of tissue-specific miRNAs; hence, it is the best choice for high-throughput detection of miRNA expressions. Jagadeeswaran et al. (2010) analyzed all candidate miRNAs of *B. mori* and found that most novel miRNAs were preferentially expressed in the pupae, whereas many of the conserved miRNAs were differentially regulated during different developmental stages. Furthermore, the expression levels of four miRNA* were remarkably higher than their corresponding miRNAs, and the expression profiles of miR and miR* were dissimilar among different developmental stages. In another study, a total of 3750 candidate miRNAs were identified using RNAfold and TripletSVM algorithms, and of these, 354 miRNAs were confirmed by microarray technology. The expression profiles of all developmental stages were analyzed using these miRNAs. The results showed that 106 miRNAs were expressed in all stages, while 248 miRNAs were egg-specific or pupa-specific, indicating that *B. mori* miRNAs may have substantial effects on embryogenesis and metamorphosis ( Zhou et al. 2008 ). Liu et al. (2010a) were the first to report spatial expression patterns of nearly 100 miRNAs in multiple normal tissues of female and male *B. mori* using microarray and northern-blotting analyses, in which only 10 miRNAs were detected to express in universal tissue types of the *B. mori,* such as bmo-let-7 and bmo-bantam, whereas the majority were distributed exclusively or preferentially in specific tissues, such as bmo-miR-275 and bmo-miR-1.

Other experimental protocols commonly used to detect miRNA expressions are polymerase chain reaction (PCR) technology, polyacryla-mide gel electrophoresis (PAGE)/northern blotting, and real-time PCR (qPCR). When all of these techniques are combined, the expressions can all be detected and depicted with much more clarity and precision, concerning exceptional miRNA expressions from specific tissues, expression characteristics of distinct miRNAs across different developmental periods, and changes in miRNA expression under special situations. Yu et al. (2008a) cloned and experimentally verified 35 *B. mori* miRNAs at 14 developmental stages, along with their expressions and distributions. Consequently, the expressions of individual miRNAs and miRNA species were markedly higher at the larva-molting stage compared with that at any other stage, indicating that miRNAs may play key regulatory roles in the *B. mori* ecdysis. Using northern blotting analysis, He et al. (2008) revealed that some *B. mori* miRNAs (e.g., bmo-miR-277) were expressed only during specific stages, indicating that these miRNAs have regulatory patterns of developmental expression. Furthermore, Yao et al. (2008) performed a reverse transcription PCR (RT-PCR) assay of four candidate miRNAs and found that these miRNAs had different expression levels among different organs, again suggesting diverse expression patterns of some miRNAs during development.

Protocols for analysis of the luciferase reporter vector are commonly used to detect the expression of miRNAs in cells. Yang et al. (2012) constructed expression vectors />ZEx-1-EGFP-pri-mir-1a/8/133 containing the promoter *ie1,* the enhancer *hr5,* and three corresponding pri-miRNA sequences. The constructed miRNA vectors were successfully transfected into BmN cells and quantitative RT-PCR (qRT-PCR) analysis showed the relative abundances of bmo-mir-1a, bmo-mir-8, and bmo-mir-133 in BmN cells.

### Prediction of *B. mori* miRNA targets

Mature miRNA 5'-end sequences (2-8 nucleotides) are complementary to the 3'-untranslated (UTR) sequences of potential target mRNAs. Furthermore, putative binding of miRNA-mRNA duplexes can form a ther-modynamically stable dimer. The use of secondary structure analysis software has become a common model for predicting target genes of *B. mori* miRNAs ( Table 1 ). He et al. 2008 predicted 1671 3'-UTR binding sites in *B. mori* genes and obtained 547 target genes, including 986 target sites, through functional conservative and binding site predictive analyses; of these, 338 target 3'-UTRs and 43 seed regions of miRNAs could form perfect base pairs. Using mFold analysis, Zeng et al. 2008 determined that the minimum free energy for hybridization was -28.2 kcal/mol between BmEm4 and bmo-mir-7 and -17.6 kcal/mol between BmEm4 and bmo-mir-79. Based on the online software RNAhybrid and MirTif, screening for the predicted binding sites, Liu et al. (2012) obtained predictions about the binding site of let-7 in the Ras 3'-UTR and found in Ras 1, Ras 2 and Ras 3 there were two, two, and five binding sites, respectively. Singh et al. (2010) predicted eight viral and 64 cellular targets of the BmNPV-encoded miRNAs by using miRanda, and the putative functions of these targets suggested that miRNAs play important roles in insect-pathogen interactions by modulating relationships between genes involved in viral replication and those involved in the host immune defence machinery. Bmo-miR-9a is a conservative miRNA. By using target prediction software RNAhybrid and RNA22, Song et al. (2013) obtained a target-binding site of Bmo-miR-9a in the 3'UTR *of Bm-ase* gene.

**Table 1.**
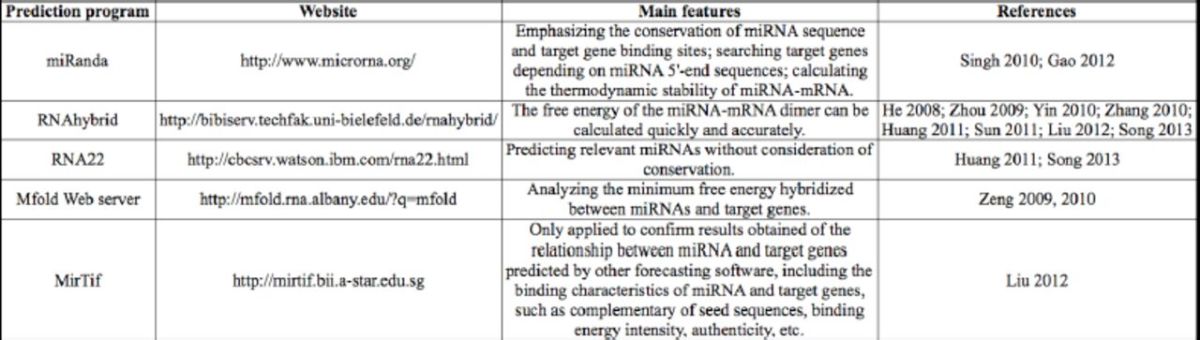
Applications of various prediction programs for *Bombyx mori* target miRNAs.

The Promega Corporation ( www.promega.com ) has developed a dual reporter gene assay system for detecting firefly luciferase and marine coelenterate luciferase. This system is used in combination with a pRL vector system in order to express the luciferase of the second reporter gene, and then the target gene expression regulated by miRNAs is investigated through the dual luciferase reporter detection system. Although this method is relatively uncomplicated, it cannot efficiently simulate the interaction between specific miRNAs and target genes either *in vivo* or in the intracellular environment. Cao et al. (2008) constructed an expression vector of the *fibroin L* chain mRNA 3'-UTR and used this vector to show that miRNAs can repress expression of the **/?***-* glucuronidase reporter gene (GUS). Sequence complementation analysis using agroinfiltra-tion followed by histochemical and biochemical assays further confirmed that four miRNAs were involved in transcriptional regulation of the specific target genes ( Table 2 ). Huang et al. (2011a) constructed a recombinant plasmid containing the targets *Fib-L* and *P25* 3'-UTR, which were co-transfected into *Sf* cells with a recombinant miRNA expression plasmid. On the basis of the relative luciferase activity, miRNA-965 and miRNA-1926 were reported to down-regulate the expression of the *Fib-L* gene via complementary combination with the target site of the target gene. Huang et al. (2011c) successfully inserted the cytoplasmic actin 3 (A3) promoter and flanked sequences of the miRNA-9a (miR-9a) precursor into a pCDNA3.0 vector to construct a recombinant plasmid, which was then transfected into BmN cells. The results of this study suggested that use of a recombinant miRNA expression vector was a favorable approach for the functional study of *B. mori* miRNAs *in vitro.* Based on these experiments, Huang et al. 2011c successfully transformed the recombinant donor plasmid, pFastBac-miR-9a, into *E. coli* DH10Bac/AcNPV and transfected it into *Sf21* insect cells with cational lipofec-tin. The results confirmed that the baculovirus expression system could be used to transcribe a recombinant vector containing miR-9a for further analysis of miR-9a function ( Huang et al. 2012a ). Similarly, Yin et al. (2010) constructed a transfection vector containing the *Bmyan* 3'-UTR and the luciferase reporter gene, which was co-transfected into a BmE cell line with bmo-miR-7, and the results of luciferase activity analysis revealed a recognition site of bmo-miR-7 on the *Bmyan* 3-UTR. Zhang et al. (2010) validated the regulating relationships between bmo-miR-7 and *Bmhairy* using similar methods; however, western blotting analyses indicated that this was potentially a fine-tuned target and not a turn-on/turn-off target. Sun et al. (2011) constructed an expression vector of the target *Fib-L* 3-UTR with the reporter gene *EGFP* and the miR-190 expression vector and transfected both of these recombinant vectors into BmN cells, which confirmed that miR-190 had a regulatory function on *Fib-L* at the cellular level. Liu et al. (2012) combined the *Ras* 3-UTR with reporter gene vectors, which were co-transfected in *Sf9* cells together with let-7 mimics. Using a dual luciferase reporter gene assay, they then predicted and verified that let-7 had target sites on the respective 3-UTRs of *Ras1, Ras2,* and *Ras* 3. To verify the regulation function of Bmo-miR-9a on the expression of *Bm-ase* gene, Song et al. (2013) constructed a Bmo-miR-9a over-expressing vector and *Bm-ase* 3'UTR fused firefly *lucif-erase* gene reporter plasmid, respectively. Then they were co-transfected into the BmN cells and the luciferase activity of co-transfected cells was suppressed compared with the control. Furthermore, the result was similar when BmN cells were co-transfected with artificial synthetic Bmo-miR-9a mimics and *Bm-ase* 3'UTR fused luciferase reporter plasmid. These results suggested that Bmo-miR-9a could down-regulate the expression of *Bm-ase* gene. Chen et al. (2013) further investigated the 3'-UTR of BmVMP23, which was destroyed by an inserted fragment, and the expression of BmVMP23 was found to be down-regulated in eggs of lethal mutant *B. mori* strain Ming *(l-e ).* Furthermore, they found a miRNA (bmo-miR-1a-3p) that matched perfectly to the 3-UTR sequence of BmVMP23 and conducted an *in vitro* co-transfection experiment to verify the expression relationship between bmo-miR-1a-3p and BmVMP23. On the basis of the evaluated luciferase activity, *luc* expression was shown to be strongly repressed, suggesting that bmo-miR-1a-3p might downregulate BmVMP23 expression via complementary interactions with target sites at the 3'-UTR.

**Table 2.**
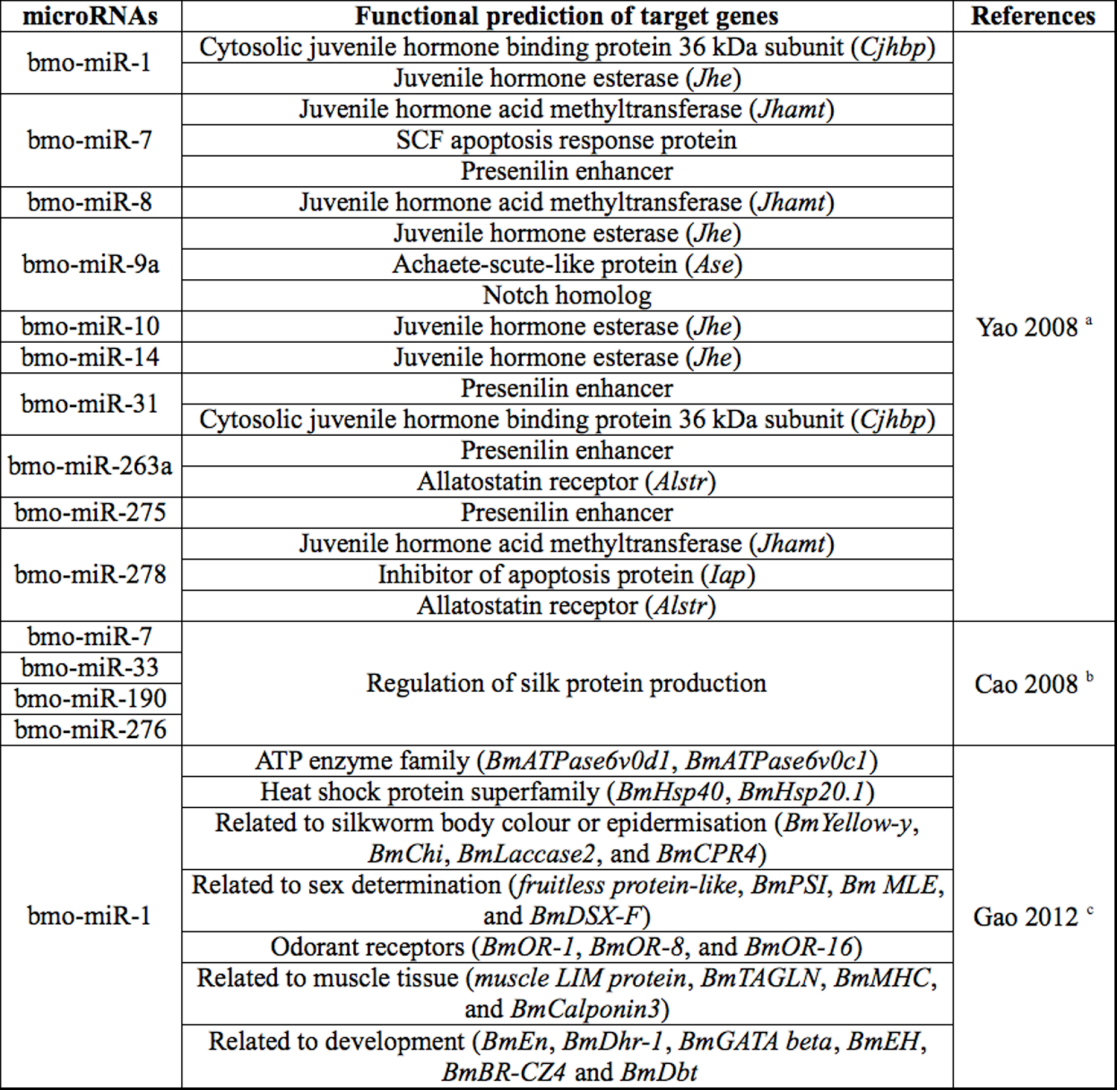
Results of predictions of several *Bombyx mori* specific miRNA target genes.

^a^ Yao searched the NCBI UniGene database to obtain all silkworm UniGenes, resulting in a total of 1 1, 198 silkworm 3'-UTR sequences and predicting 10 potential target genes related to silkworm miRNAs.

^b^ Cao found at least four miRNA binding sites in the silkworm *Fib-L* using target gene locus prediction software.

^c^ Gao predicted 87 potential bmo-miR-1 target genes based on available *Bombyx mori* 3'-UTR data *in silico.*

### Functional studies of *B. mori* miRNAs

Yu et al. (2008b) cloned and predicted target genes of miRNAs based on gene ontology (GO) and Kyoto Encyclopedia of Genes and Genomes (KEGG) functional classifications and found that more than half of the target genes were involved in protein binding, metabolism, cell processes, as well as catalytic activity. Most of these genes were also found to be related to amino acid metabolism, glucose metabolism, signal transduction, energy metabolism, endocrine, tumors, and protein processing and translation pathways. Furthermore, this study revealed that the species and number of miRNAs were both much higher at the 4 instar molting period than at other developmental stages, indicating that miRNAs may play significant roles in the molting phase. The results of the target prediction study by Cai et al. (2010) clearly indicated that a large number of *B. mori* miRNAs modulated more than one component of hormone signaling pathways and/or hormone biosynthesis-related proteins, which implied that they have considerable effects in *B. mori* developmental processes. Similarly, Yao et al. (2008) indicated that the 10 targets regulated by miRNAs were all involved in important life activities in the *B. mori.* As shown in Table 2 , the different target genes identified indicate that miRNAs could play extremely emphatic roles in a variety of different physiological intracellular activities ( Yao et al. 2008 ). Sun et al. (2011) investigated the tissue-specific expressions of miR-33, miR-7, and miR-190 and found that all three of these miRNAs were expressed in the *B. mori* posterior silk gland (PSG) at the 5 instar larval stage. In particular, the expression level of miR-190 was increased in the PSG relative to other tissues, suggesting that miR-190 may be involved in regulating the expression of the silk protein. Singh et al. (2012) provided evidence that the BmNPV modulated the small-RNA-mediated defense of its host by encoding an miRNA (bmnpv-miR-1) that down-regulated the expression of the host GTP-binding nuclear protein, Ran. Furthermore, this study revealed that the immediate-early gene targeted by bmo-miR-8 could be controlled by bmnpv-miR-1 and Ran dsRNA, resulting in increasing virus infection levels in the *B. mori* larvae. Previously, in a computational prediction of the viral and cellular targets of BmNPV virus-encoded miRNAs, Singh et al. (2010) discovered that these viral miRNAs played significant roles in insect-pathogen interactions by regulating viral replication-related and host immune defense-related genes. Zhou et al. (2009) induced BmN-SWU1, BmN-SWU2, and BmE cells with different concentrations of ecdysone and then detected the expression levels of bmo-1et-7. The results demonstrated distinctive induction effects, and furthermore, both the cellular type and induction time had extreme impacts on the induction effect. In the program to figure out the intercommunication between miR-1 and *B. mori* molting disorder, when prepupal stage *B. moris* were injected with miR-1 mimics, the old epidermis was not slough off, and the formation of the internal pupal epidermis was slightly affected by the old translucent epidermis ( Gao et al. 2012 ). This result indicated that up-regulation of Bmo-miR-1 may lead to *B. mori* molting disorder.

### Non-canonical functions of miRNAs

MiRNAs are small ncRNAs that are involved in post-transcriptional gene regulation. According to the canonical model, miRNAs can recognize protein-coding mRNAs and exert their roles predominantly in the cytoplasm. However, several studies have revealed that miRNAs can also be transported from the cytoplasm to the nucleus ( Meister et al. 2004 ; Politz et al. 2006 ; Hwang et al. 2007 ). Recently, Chen (2013) highlighted that several nuclear miRNAs can act in a non-canonical manner to regulate the biogenesis and function of ncRNAs, including miRNAs and long ncRNAs ( Hansen et al. 2011 ; Zisoulis et al. 2012 ; Tang et al. 2012 ). MiRNAs determine the repression of translational processes or the degradation of mRNA targets. Another mode of miRNA-mediated regulation of translation repression or activation has been reported, which involves the binding of miRNA to the 5'-UTR of its target gene ( Lytle et al. 2007 ; Ørom et al. 2008 ; Tsai et al. 2009 ). Letizia and Masotti (2013) recently reviewed the possible interactions and action mechanisms based on the results of several studies and discussed the bioinformatics tools and public databases currently available for predicting miRNA binding sites in the 5 '-UTR.

### Concluding remarks and future directions

*Bombyx mori* is a lepidopteran insect of economic importance. Since its genome was first sequenced, functional genomics research has revealed information about the sophisticated mechanisms regulating gene expression, with both applied and theoretical significance. After summarizing research related to the discovery and identification protocols of *B. mori* miRNAs, we discovered that the majority of researchers prefer to use high-throughput sequencing protocols for seeking and obtaining candidate miRNAs from *B. mori.* As the number of such studies continues to increase, the next challenges will be to ensure efficient and accurate access to candidate miRNAs. Some researchers focus on bioinformatics prediction tools to obtain a large number of *B. mori* miRNAs, which further encourages more efficient and strict requirements in the development of bioinformatics and computer software programs.

Methods for analysis and verification of silkworm miRNA expression profile are identical to those for other species-cloning and library construction, high-throughput expression profiling, RT-PCR, or northern blotting, etc. Stem-loop RT-PCR is known to be a very sensitive method for the quantitative determination of known miRNAs. Gao et al. (2012) designed two groups of primers to identify conserved miRNAs in the non-model insect, *Spodoptera litura* . One group of primers differed from those frequently used in silkworm studies, particularly with respect to the forward primer. When utilizing the more-specific miRNA forward primer, agarose gel electro-phoresis revealed that the fragments were displayed by a single band and no nonspecific fragment was amplified. Therefore, this method could be used to amplify specific silkworm miRNAs. Currently, PCR and northern blotting are widely used to detect individual miRNAs. Although PCR is a highly sensitive method, the high incidence of false-positives and difficulties of primer design have generally limited its use in this field. In addition, traditional northern blotting protocols are relatively complicated, time-consuming, and inconvenient. Li et al. (2012) reported a novel method for detecting individual miRNAs that was rapid but required specific liquid hybridization and color development (LHCD) instruments for detection of fluorescent signals. Based on the rapidity of liquid hybridization and signal amplification for detection of the avidin-biotin complex (ABC), LHCD can identify a one-nucleotide difference within a miRNA family and allows sensitive detection of 2.5 fmol of miRNAs. So, LHCD is a convenient and efficient alternative method for miRNA analyses ( Li et al. 2012 ).

Shamayra et al. (2006) investigated the posterior silk gland of the *B. mori* Nistari strain and obtained five U6 small nuclear RNA (snRNA) isoforms. Their sequences were identical to the 35 full-length U6 variants recently released in the Whole Genome Shotgun (WGS; Mita et al. 2004 ) database of the p50T strain. The main function of U6 snRNA is in pre-mRNA processing, in which variants can modulate the assemblage of the catalytic core as well as affect the splicing rate ( Shamayra et al. 2006 ). U6 snRNA is commonly employed as the universal reference gene in identifying gene expression ( Liu et al. 2008 ; Yu et al. 2008a ; Yu et al. 2009 ; Huang et al. 2011b ).

With respect to target gene prediction analyses of miRNAs, gene prediction software is most commonly employed ( Table 1 ). To date, no miRNA analysis software specifically for silkworms has been developed, necessitating further experiments to verify the prediction results. Currently, the most frequently used method for investigation of miRNA functions involves identification of the miRNA through inhibiting the target gene at the cellular level. In this respect, the *B. mori* ( *Bm* ) and *Spodoptera frugiperda* ( *Sf* ) cell lines are commonly used for transfection in medium, and the *Ag-robacterium* infection method has also been applied ( Cao et al. 2008 ). Both single and dual fluorescence expression systems are generally used to investigate miRNA expression and the relationship between a miRNA and its target gene. However, different promoters of recom-binant vectors could affect transient expression. Huang et al. (2012b) used recom-binant plasmids containing six different promoters for transfection, and transcriptional experiments revealed that the three promoters of *Fib-H* , *A3* , and *IE-1* sequentially strengthened transient expression of the *luc* reporter gene in BmN cells. Although such authentications at the cellular level can demonstrate whether miRNAs may negatively impact target genes at the post-transcriptional level or confirm that there are binding sites of miR-NAs on the 3ʹ-UTR of targets, further confirmation of these results is also necessary. In all relevant literature surveyed, there was no indication of miRNAs up-regulating their targets in the silkworm. However, the up-regulation effect has been reported in human miRNAs ( Vasudevan et al. 2007 ). At present, several studies have emerged from India and China regarding baculovirus or microsporidian miRNAs in *B. mori* , which presents a new direction for research concerning viral or bacterial gene functions ( Dang et al. 2009 ; Chen et al. 2010; Singh et al. 2010 ; Shirina et al. 2011 ).

As key factors in post-transcriptional gene regulation, miRNAs are well known to have very important regulatory functions in significant biological processes such as cellular differentiation, proliferation, apoptosis, development, and disease occurrence. Basic research aimed directly at silkworm miRNAs has demonstrated that miRNAs can produce marked effects on the mechanisms underlying the developmental processes of silkworms. In addition, investigations conducted thus far have laid the foundation for improving our understanding of RNA regulation networks and the molecular mechanisms involved in gene expression patterns throughout different life stages. MiRNA research represents important progress in the study of ncRNAs and may provide further information on the activities of as yet unknown ncRNAs.

## Abbreviations

BmNPV: *Bombyx mori* nucleopolyhedrosis virus
miRNA: microRNA
ncRNA: non-coding RNA
